# Effects of alpha 2-adrenoceptor agonists dexmedetomidine and guanfacine on morphine analgesia and tolerance in rats

**DOI:** 10.3109/03009734.2011.597889

**Published:** 2011-10-29

**Authors:** Sinan Gursoy, Ercan Ozdemir, Ihsan Bagcivan, Ahmet Altun, Nedim Durmus

**Affiliations:** ^1^Anesthesiology and Reanimation, Cumhuriyet University School of Medicine, Sivas, Turkey; ^2^Department of Physiology, Cumhuriyet University School of Medicine, Sivas, Turkey; ^3^Department of Pharmacology, Cumhuriyet University School of Medicine, Sivas, Turkey

**Keywords:** Dexmedetomidine, guanfacine, morphine, α_2_-receptor agonist, tolerance

## Abstract

**Background:**

Alpha 2 (α_2_)-adrenoceptor agonists may be useful for their potential to increase or prolong opioid analgesia while attenuating the development of opioid tolerance. The purpose of this study was to investigate the effects of dexmedetomidine and guanfacine (α_2_-adrenoceptor agonists) on morphine analgesia and tolerance in rats.

**Methods:**

Adult male Wistar albino rats weighing 195–205 g were used. To constitute morphine tolerance, animals received morphine (50 mg/kg) once daily for 3 days. After the last dose of morphine had been injected on day 4, morphine tolerance was evaluated by analgesia tests. The analgesic effects of dexmedetomidine (20 μg/kg), guanfacine (0.5 mg/kg), MK-467 (0.25 mg/kg), and morphine were estimated at 30-min intervals (0, 30, 60, 90, and 120 min) by tail-flick and hot-plate analgesia tests.

**Results:**

Our findings indicate that dexmedetomidine and guanfacine attenuated the expression of morphine tolerance. In addition, administration of dexmedetomidine with morphine increased morphine analgesia. On the contrary, data suggested that MK-467 (an α_2_-adrenoceptor antagonist) decreased morphine analgesia and increased morphine tolerance in analgesia tests.

**Conclusion:**

In conclusion, we observed that co-injection of dexmedetomidine or guanfacine with morphine attenuated the expression of tolerance to the analgesic effect of morphine and that dexmedetomidine enhanced the morphine analgesia.

## Introduction

Morphine and related opioid drugs produce potent analgesia by activating specific opioid receptors expressed in brain, spinal cord, and peripheral tissues. Chronic administration of these drugs, however, leads to the development of both tolerance and dependence, limiting the utility of opioids in the management of severe pain syndromes. The mechanisms underlying the development of tolerance are complex and not completely understood. There are several possible explanations for the development of opioid tolerance, including opioid peptide receptor desensitization, up-regulation of the cAMP pathway (supersensitization of adenylyl cyclases, receptor coupling with stimulatory G-proteins), induction of nitric oxide (NO)-cGMP systems, and protein kinase-dependent neuroadaptative changes in signal transduction cascades (second-messenger-dependent protein kinases and G-protein-coupled receptor kinases) (1–3). We also recently demonstrated that the nitric oxide–cGMP signal pathway plays a pivotal role in developing tolerance to the analgesic effect of morphine (4).

The noradrenergic system plays an important role in opioid actions. Alpha 2 (α_2_)-adrenoceptor agonists potentiate morphine analgesia, and there is analgesic synergism between endogenous α_2_-noradrenergic and opioidergic mechanisms in the spinal cord of mice and rats (5,6). Substance P-induced behavioral nociceptive responses are inhibited by intrathecally administered opioid agonists and α_2_-adrenoceptor agonists in mice (7). In addition, the α_2_-adrenoceptor antagonist, yohimbine, attenuates the analgesic effects of opioid agonists in the rat (8). Three α_2_-adrenoceptor subtypes have been identified in humans and in mice, i.e. α_2A_-, α_2B_-, and α_2C_-adrenoceptors. All three are coupled to Gi/o-type G-proteins, and their activation induces similar second-messenger responses as those mediated by opioid peptide receptor activation, i.e. inhibition of adenylyl cyclases, activation of hyperpolarizing K^+^ channels, and inhibition of Ca^2+^ channels. In the central nervous system (CNS), presynaptically localized α_2_-adrenoceptors inhibit the release of noradrenaline and several other neurotransmitters. The α_2A_-adrenoceptor is the principal α_2_-adrenoceptor subtype found in the CNS. The analgesic and sedative properties of α_2_-adrenoceptor agonists are mainly attributed to α_2A_-adrenoceptor activation, as evidenced by recent studies on mice lacking functional α_2A_-adrenoceptors (9,10).

Dexmedetomidine is a subtype-non-selective α_2_-adrenoceptor agonist. It has potent sympatholytic, analgesic, sedative, and anesthetic effects in animals (11–13) and in humans (14), and it is in clinical use as a sedative agent in the intensive care setting (15). Dexmedetomidine decreases the turn-over of the monoamine neurotransmitters noradrenaline, dopamine, and serotonin (5-HT) in brains of rats (16), but this effect was absent in mice lacking α_2A_-adrenoceptors. Also, other typical α_2_-adrenoceptor-mediated effects of dexmedetomidine, such as inhibition of locomotor activity and hypothermia, were very markedly attenuated, but not totally abolished, in α_2A_-adrenoceptor-deficient mice (11). The alpha-2 agonist guanfacine does not discriminate between the three subtypes of alpha-2 receptors, although guanfacine has somewhat higher affinity for the 2A versus 2B and 2C subtypes (17).

Rapid development of tolerance limits the usefulness of morphine and other potent opioids in long-term treatment. Morphine tolerance is a complex phenomenon, involving also the brain noradrenergic system (18). There is much evidence that the brain noradrenergic system is involved in many of the actions of morphine (19,20), but the role of α_2_-adrenoceptors on the mechanisms of development of tolerance to morphine are still unclear. Thus, the aim of the present study was to determine the effects of dexmedetomidine and guanfacine, as α_2_-adrenoceptor agonists, on morphine analgesia and tolerance in rats.

## Materials and methods

### Animals

The experiments were performed on adult male Wistar albino rats weighing 195–205 g. Animals were housed four per cage in a room maintained at 22°C ± 1°C with an alternating 12 h dark/12 h light cycle and free access to water and food. Animals were acclimatized to laboratory conditions before the test. All experiments were carried out blindly between 09.00 and 17.00 h. The experimental protocols were approved by the Cumhuriyet University Animal Ethics Committee (license number: 87/Ethic).

### Drugs

Dexmedetomidine, guanfacine, MK-467 (Sigma-Aldrich, USA), and morphine sulphate (Cumhuriyet University Hospital, Turkey) were dissolved in physiological saline. Solutions were freshly prepared on the days of experimentation. Subcutaneous (s.c.) morphine (5 mg/kg), intraperitoneal (i.p.) dexmedetomidine (20 μg/kg), guanfacine (0.5 mg/kg), and MK-467 (0.25 mg/kg) were administered before the analgesia tests.

### Induction of morphine tolerance

To constitute morphine tolerance, a 3-day cumulative dosing regimen was used. The treatment schedule consisted of twice daily s.c. doses of morphine given at 30 mg/kg (a.m.) and 45 mg/kg (p.m.) on day 1; 60 and 90 mg/kg on day 2; and 120 mg/kg twice on day 3. Animals were assessed for tolerance on the 4th day, as described by Way et al. (21). Tolerance was assessed based on loss of the antinociceptive effects of a test dose (5 mg/kg; s.c.) of morphine. On day 4, tail-flick and hot-plate tests were done for each rat to average them as a base-line latency; then a challenge dose of morphine (5 mg/kg; s.c.) was injected; 30 min after the morphine injection further tail-flick and hot-plate tests were performed for evaluations of the development of tolerance to morphine.

In saline-treated rats, saline was administered twice daily for 3 days according to the same injection schedule.

### Antinociceptive tests

To evaluate thermal nociception, we used a standardized tail-flick apparatus (May TF 0703 Tail-flick Unit; Commat, Turkey). The radiant heat source was focused on the distal portion of the tail at 3 cm after administration of the vehicle and study drugs. Following vehicle or compound administration, tail-flick latencies (TFL) were obtained. The infrared intensity was adjusted so that basal TFL occurred at 2.9 ± 0.5 s. Animals with a base-line TFL below 2.4 or above 3.4 s were excluded from further testing. The cut-off latency was set at 15 s to avoid tissue damage. Any animal not responding after 15 s was excluded from the study. The hyperalgesic response in the tail-withdrawal test is generally attributed to central mechanisms (22,23).

A second analgesia test was that of hot-plate testing. In this test, animals were individually placed on a hot-plate (May AHP 0603 Analgesic Hot-plate; Commat, Turkey) with the temperature adjusted to 55°C ± 0.5°C. The latency to the first sign of paw licking or jump response to avoid the heat was taken as an index of the pain threshold; the cut-off time was 30 s in order to avoid damage to the paws. The antinociceptive response on the hot-plate is considered to result from a combination of central and peripheral mechanisms (22).

### Experimental protocols

Antinociceptive effects of dexmedetomidine, guanfacine, MK-467, and morphine were estimated at 30-min intervals (0, 30, 60, 90, and 120 min) by tail-flick and hot-plate tests in rats (*n* = 7–8). After induction of morphine tolerance, the analgesic response to the challenge dose was determined again on day 4 at 30-min intervals after the same morphine (5 mg/kg challenge dose; s.c.) injection as on the first day. To evaluate the effects of dexmedetomidine, guanfacine, and MK-467 on expression of morphine tolerance, morphine-tolerant animals received dexmedetomidine (20 mg/kg; i.p.), guanfacine (0.5 mg/kg; i.p.), or MK-467 (0.25 mg/kg; i.p.). In the saline-treated group, animals received saline (10 mL/kg) instead of morphine.

### Data analysis

In order to calculate percent maximal antinociceptive effects (% MPE), tail-withdrawal latencies (tail-flick) and lick/escape latencies (hot-plate) were converted to percent antinociceptive effect using the following equation (equation 1):

(Eq.1) $$\begin{array}{l} \%\;\mathrm{MPE}\;=\;[(\mathrm{test}\;\mathrm{latency}-\mathrm{base}\text{​}-\text{​}\mathrm{line})\text{​ ​​}/\\ (\mathrm{cut}\text{​}-\text{​}\mathrm{off}-\mathrm{base}\text{​}-\text{​}\mathrm{line})]\;\times \;100. \end{array}$$

### Statistical analysis

Antinociceptive effects of the drugs were measured as tail-flick and hot-plate latencies in all groups for each rat and converted to % MPE. The data were analyzed by analysis of variance (ANOVA) followed by Tukey test. All data are presented as means ± SEM. The level of significance was set at *p* < 0.05.

## Results

### Effects of dexmedetomidine on morphine analgesia and tolerance

**Figure 1.**
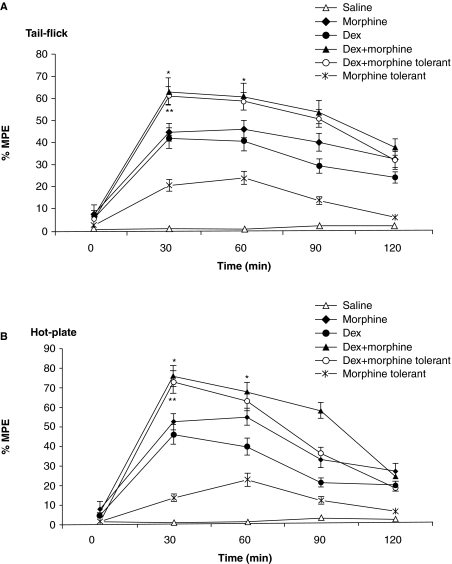
Effects of dexmedetomidine (DEX; 20 μg/kg; i.p.) on morphine analgesia and tolerance in tail-flick (A) and hot-plate (B) tests. Each point represents the mean ± SEM of percent of maximal possible effect (% MPE) for 8 rats. *p < 0.05 compared to morphine group, **p < 0.01 compared to morphine-tolerant group.

Tukey's test indicated that pretreatment of animals with dexmedetomidine reduced (increased mean of % MPE value) the expression of tolerance to the morphine antinociceptive effect in both tail-flick (*p* < 0.01; Figure 1A) and hot-plate tests (*p* < 0.01; Figure 1B). In addition to this, administration of dexmedetomidine with morphine increased the antinociceptive effect of morphine in tail-flick (*p* < 0.05; Figure 1A) and hot-plate tests (*p* < 0.05; Figure 1B). The peak value was observed at 30 min after administration of morphine and dexmedetomidine in analgesia tests (tail-flick: 62.70 ± 5.6 and hot-plate: 75.40 ± 5.9). Furthermore, these data suggested that dexmedetomidine alone has got an analgesic effect (*p* < 0.05).

### Effects of guanfacine on morphine analgesia and tolerance

**Figure 2.**
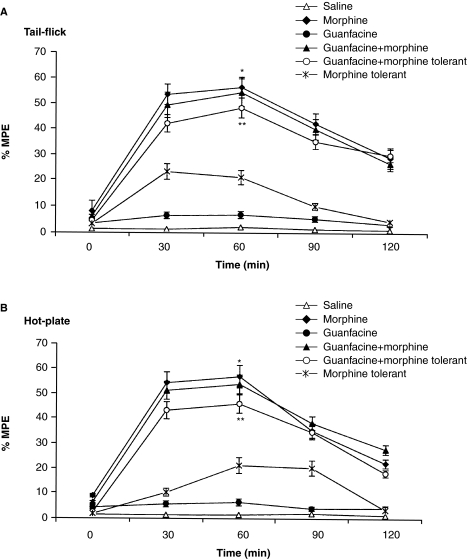
Effects of guanfacine (0.5 mg/kg; i.p.) on morphine analgesia and tolerance in tail-flick (A) and hot-plate (B) tests. Each point represents the mean ± SEM of percent of maximal possible effect (% MPE) for 7 rats. *p < 0.01 compared to saline group, **p < 0.05 compared to morphine-tolerant group.

It was found that guanfacine reduced the expression of tolerance to the morphine analgesic effect in tail-flick (*p* < 0.01; Figure 2A) and hot-plate tests (*p* < 0.01; Figure 2B). However, administration of guanfacine did not increase the antinociceptive effect of morphine in tail-flick (Figure 2A) and hot-plate (Figure 2B) tests. In their analgesia tests the peak value of the morphine group was observed after 60 min. Furthermore, there was no evidence to suggest that guanfacine alone had an analgesic effect.

### Effects of MK-467 on morphine analgesia and tolerance

**Figure 3.**
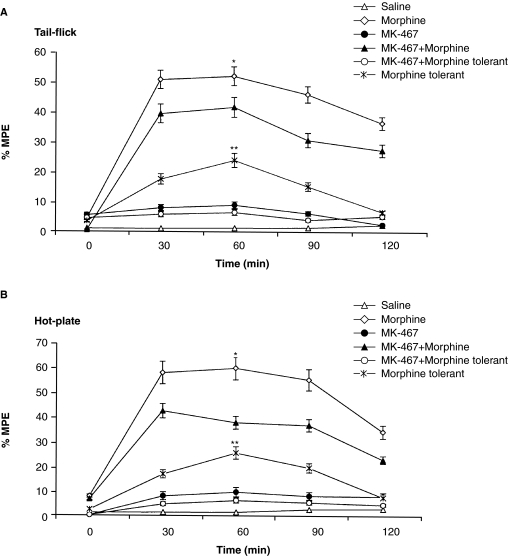
Effects of MK-467 (0.25 mg/kg; i.p.) on morphine analgesia and tolerance in tail-flick (A) and hot-plate (B) tests. Each point represents the mean ± SEM of percent of maximal possible effect (% MPE) for 7 rats. *p < 0.05 compared to MK-467 + morphine group, **p < 0.01 compared to MK-467 + morphine-tolerant group.

Systemic administration of MK-467 (α_2_-adrenoceptor antagonist) together with morphine produced a decrease in % MPE in both the tail-flick (*p* < 0.01; Figure 3A) and hot-plate (*p* < 0.01; Figure 3B) assays when compared to rats given morphine only. Pretreatment of animals with MK-467 increased the expression of tolerance to the morphine antinociceptive effect in tail-flick and hot-plate tests (Tukey; *p* < 0.05 and *p* > 0.05, respectively). Also, MK-467 alone had no analgesic effect.

### Analgesic effects of different doses of dexmedetomidine

**Figure 4.**
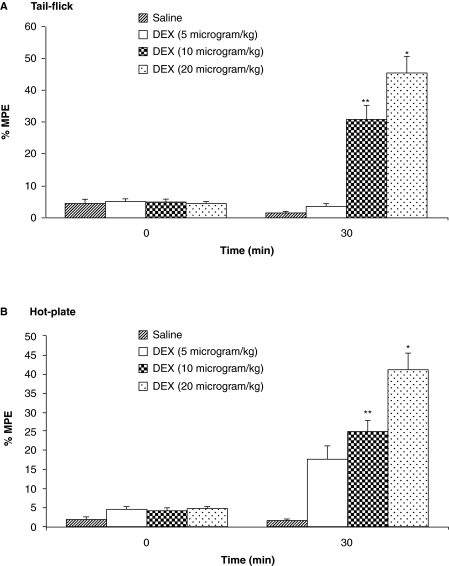
Analgesic effects of different doses of dexmedetomidine (5, 10, and 20 μg/kg; i.p.) as measured in tail-flick (A) and hot-plate (B) tests. Each point represents the mean ± SEM of percent of maximal possible effect (% MPE) for 8 rats. *p < 0.001, **p < 0.01 compared to saline-treated group.

In dose–response studies of the dexmedetomidine effect, the analgesic response was measured for three different doses of dexmedetomidine (5, 10, and 20 mg/kg; i.p.) at 30-min intervals (0 min and 30 min) by tail-flick and hot-plate tests. The maximum analgesic effect was observed after administration of 20 mg/kg dexmedetomidine (45.50 ± 6.90 for tail-flick test and 41.30 ± 7.13 for hot-plate test). The % MPE produced by dexmedetomidine (20 mg/kg) was higher than in the other groups (*p* < 0.01; Figure 4A) and hot-plate test (*p* < 0.01; Figure 4B).

## Discussion

In this study, we demonstrated that the α_2_-adrenoceptor agonists dexmedetomidine and guanfacine play a significant role in morphine analgesia and tolerance. Obtained data suggested that co-injection of morphine with dexmedetomidine increased the analgesic effects of morphine and attenuated the development of tolerance to morphine analgesia. Similarly, the data showed that guanfacine reduced the expression of tolerance to the analgesic effect of morphine. On the other hand, the α_2_-adrenoceptor antagonist MK-467 decreased the analgesic effects of morphine and increased the expression of tolerance to morphine. All these results supported the idea that the α_2_-adrenoceptor agonists attenuate development of tolerance to morphine analgesia.

Pain modulation is a dynamic process, which includes many interactions among complex ascending and descending neuronal systems (24,25). Opioidergic and noradrenergic pathways have very important roles in analgesia and tolerance (26,27). Activation of opioid and α_2_-adrenergic receptors inhibits the transmission of pain sensation at spinal and supraspinal levels. While α_2_-adrenoceptors inhibit nociception and have analgesic synergy with opioids, there is evidence that α_1_-adrenoceptors may facilitate nociception and oppose opioid analgesia. Microinjection of the α_1_-adrenoceptor antagonist, prazosin, into the nucleus raphe magnus, a brain nucleus involved in nociceptive processing, produces antinociception similarly to the α_2_-adrenoceptor agonist clonidine (28). It has also been reported that prazosin augments clonidine analgesia in amphibians (29). Morphine microinjection into the ventrolateral periaqueductal gray inhibits nociceptive tail responses, an effect mediated by acetylcholine, 5-HT, and α_2_-adrenergic receptors in the spinal cord. In some experiments, however, microinjection of morphine into the periaqueductal gray has also facilitated nociceptive responses; this facilitation of hot-plate responses was mediated by α_1_-adrenoceptors in the spinal cord (30). Noradrenaline has an important role in the modulation of pain transmission especially at the level of the spinal cord (31). In accordance with our findings, Belgrade et al. (32) informed that the administration of α_2_-adrenoceptor agonists increased the morphine antinociceptive effect in patients. Intrathecal or direct spinal injection of noradrenaline produces antinociception and decreased dorsal horn neuronal activity. However, noradrenaline has two directional modulatory effects on nociception. Activation of α_1A_- but not α_1B/D_-adrenoceptors mediates potentiation of spinal nociceptive reflexes (33). Noradrenaline enhances motoneuronal responses to stimulation of nearby ventral interneurons via activation of α_1A_-adrenoceptors (34). The principal adrenoceptor subtype mediating noradrenaline antinociception has recently been identified as the α_2A_-adrenoceptor based on studies in gene-targeted mice (11). In addition to this, α_2C_-adrenoceptors may also contribute to spinal analgesia (35). Increased central noradrenergic activity potentiates morphine analgesia (36), but acute morphine administration inhibits noradrenergic neurons in the locus coeruleus and reduces noradrenaline release in its projection areas (37). Some centrally acting sympathomimetic agents, such as ephedrine, which act primarily through enhancing the release of stored catecholamines, potentiate morphine analgesia, although they do not have analgesic effects when given alone (19,38).

The mechanisms of opioid tolerance comprise changes at the receptor level as well as at downstream sites (39). Adaptational changes directly affecting the receptors involve their phosphorylation by G-protein-coupled receptor kinases (GRK) and subsequent binding of β-arrestin, resulting in uncoupling of the receptor from its associated G-proteins (receptor desensitization). Subsequent to receptor uncoupling, cell surface-located receptors may become internalized and either dephosphorylated and recycled back to the cell surface (resensitization), or targeted to lysosomes for degradation (40). Accordingly, uncoupling and internalization effectively contribute to desensitization of opioid peptide receptor signaling and, thus, to the phenomenon of opioid tolerance. In addition, there are also other alterations of opioid peptide receptor signaling. Acute administration of opioid agonists decreases cellular cAMP levels, but prolonged treatment triggers up-regulation of adenylyl cyclase activity (41). This may be one of the cellular adaptations that underlie neuronal hyperactivity during opioid tolerance and withdrawal (42). The μ-opioid peptide receptor response to the antinociceptive drug morphine can be differently regulated at different levels of the pain perception system. It appears that β-arrestin-mediated mechanisms of desensitization are major contributors to the development of tolerance at both supraspinal and spinal sites. Elimination of β-arrestin-mediated mechanisms by gene targeting has revealed that a protein kinase C (PKC)-mediated mechanism also contributes to the development of morphine tolerance at the spinal level (43).

The interactions between central noradrenergic and opioidergic mechanisms are complex. The localization of and the receptor subtypes involved in the interactions of these systems with regard to antinociception and analgesic tolerance are still unclear. However, the obtained data suggested that dexmedetomidine increased the antinociceptive effect of morphine. In addition, we also determined that the α_2_-adrenoceptor agonists dexmedetomidine and guanfacine attenuated the expression of tolerance to the analgesic effect of morphine.
